# Von Hippel–Lindau (VHL) small-molecule inhibitor binding increases stability and intracellular levels of VHL protein

**DOI:** 10.1016/j.jbc.2021.100910

**Published:** 2021-06-24

**Authors:** Julianty Frost, Sonia Rocha, Alessio Ciulli

**Affiliations:** 1 Division of Biological Chemistry and Drug Discovery, School of Life Sciences, University of Dundee, Dundee, Scotland, United Kingdom; 2 Center for Gene Regulation and Expression, School of Life Sciences, University of Dundee, Dundee, Scotland, United Kingdom; 3 Department of Molecular Physiology and Cell Signalling, Institute of Systems, Molecular and Integrative Biology, University of Liverpool, Liverpool, United Kingdom

**Keywords:** E3 ubiquitin ligase, small molecule, inhibitor, proteolysis, proteostasis, hypoxia, hypoxia-inducible factor, von Hippel–Lindau, chemical probe, AMY1, amylase 1, CRL2^VHL^, Cullin2 RING E3 ligase complex, DMSO, dimethyl sulfoxide, HFF, human foreskin fibroblast, HIF-α, hypoxia-inducible factor-alpha, O_2_, oxygen, PHD, prolyl-hydroxylase, PROTAC, proteolysis-targeting chimera, TMT, tandem mass tag, VHL, Von Hippel–Lindau

## Abstract

Von Hippel–Lindau (VHL) disease is characterized by frequent mutation of VHL protein, a tumor suppressor that functions as the substrate recognition subunit of a Cullin2 RING E3 ligase complex (CRL2^VHL^). CRL2^VHL^ plays important roles in oxygen sensing by targeting hypoxia-inducible factor-alpha (HIF-α) subunits for ubiquitination and degradation. VHL is also commonly hijacked by bifunctional molecules such as proteolysis-targeting chimeras to induce degradation of target molecules. We previously reported the design and characterization of VHL inhibitors VH032 and VH298 that block the VHL:HIF-α interaction, activate the HIF transcription factor, and induce a hypoxic response, which can be beneficial to treat anemia and mitochondrial diseases. How these compounds affect the global cellular proteome remains unknown. Here, we use unbiased quantitative MS to identify the proteomic changes elicited by the VHL inhibitor compared with hypoxia or the broad-spectrum prolyl-hydroxylase domain enzyme inhibitor IOX2. Our results demonstrate that VHL inhibitors selectively activate the HIF response similar to the changes induced in hypoxia and IOX2 treatment. Interestingly, VHL inhibitors were found to specifically upregulate VHL itself. Our analysis revealed that this occurs *via* protein stabilization of VHL isoforms and not *via* changes in transcript levels. Increased VHL levels upon VH298 treatment resulted in turn in reduced levels of HIF-1α protein. This work demonstrates the specificity of VHL inhibitors and reveals different antagonistic effects upon their acute *versus* prolonged treatment in cells. These findings suggest that therapeutic use of VHL inhibitors may not produce overt side effects from HIF stabilization as previously thought.

The von Hippel–Lindau (VHL) tumor suppressor is a multisubunit Cullin RING E3 ligase (CRL2^VHL^)—composed of Cullin2 as the central scaffold subunit, Rbx1 as RING subunit, ElonginB and ElonginC as adaptor subunits, and VHL as substrate recognition subunit (1, 2). VHL functions to specifically bind hydroxylated hypoxia-inducible factor 1-alpha (HIF-1α), mediating polyubiquitination and subsequent targeting of HIF-1α for proteasomal degradation (3). HIFs are transcription factors that regulate the response to reduced oxygen (O_2_) availability termed hypoxia. HIF is composed of an O_2_-insensitive β-subunit (HIF-β) that is stable, and an O_2_-labile α subunit, of which three isoforms are known: HIF-1α, HIF-2α, and HIF-3α. The recognition and ubiquitination of HIF-α subunits by VHL is dependent on proline hydroxylation of the O_2_-dependent degradation domain of HIF-α, a post-translational modification mediated by 2-oxoglutarate, iron (II) dioxygenases called prolyl-hydroxylases (PHDs). Proline hydroxylation results in a high-affinity binding of HIF-α to VHL and thus subsequent ubiquitination and proteasomal degradation of HIF-α under normal O_2_ conditions (3).

Mutations in human VHL result in a number of abnormalities, collectively called VHL disease when occurring in the germ line (4). However, VHL is also often loss in clear cell renal cell carcinoma (5). The fact that VHL is a tumor suppressor protein, and that VHL loss is found in clear cell renal cell carcinoma tumors, have led to the hypothesis that VHL would not be a good target to inhibit and that chronic HIF stabilization by VHL inhibitors might have detrimental side effects (6). Apart from HIF, other substrates for VHL have been postulated (7). These include Aurora A, ZHX2, NDRG3, and B-Myb (8, 9, 10, 11). Understanding VHL functions and the pharmacology of HIF stabilizers and hypoxia inducers is thus important in a variety of physiological and pathological conditions (12).

We had previously developed several compounds, able to selectively bind to and inhibit VHL (13, 14). Of these, VH298 is a potent chemical probe that triggers the hypoxic response *via* a different mechanism to other HIF stabilizers, that is, by blocking the VHL:HIF-α protein–protein interaction downstream of HIF-α hydroxylation (13). We have also characterized the activity of a related compound VH032 (15), in its ability to induce the transcriptional response to hypoxia and how this compares to hypoxia (1% O_2_) or PHD inhibition using an unbiased RNA-Seq approach (16). VH298 has also been used to trigger the hypoxic response in mice, demonstrating its appropriateness for *in vivo* applications (17, 18). In distinct applications, VHL ligands are widely used as part of heterobifunctional degrader molecules known as proteolysis-targeting chimeras (PROTACs) (19, 20, 21). We and others have extensively demonstrated the use of VHL ligands VH032 and VH298 in PROTACs targeting Bromodomain and extra-terminalproteins (22, 23, 24), protein kinases (25, 26, 27) among many other target proteins, including E3 ligases themselves as demonstrated by VHL homo-PROTACs (28).

Given the widespread use and applications of VHL ligands, inhibitors, and VHL-based PROTACs, it is important to understand the effect of VHL inhibition to the intracellular proteome in an unbiased fashion. Here, we investigate how VHL inhibitors alter the proteome of cells, using quantitative tandem mass tag (TMT) labeling–based MS. We compare these alterations to those occurring after hypoxia or exposure to a 2-oxoglutarate dioxygenase inhibitor, IOX2. We show that VH032 and its more potent related compound, VH298, increase VHL protein levels—an increase that is not observed following hypoxia or IOX2 treatment. Increases in VHL protein levels are due to stabilization of specific VHL isoforms and not alteration of mRNA. VHL protein increases result in reduction of HIF levels following prolonged VH298 treatment in cells.

## Results

### Proteomic analysis of different HIF stabilizing agents

**Figure 1:**
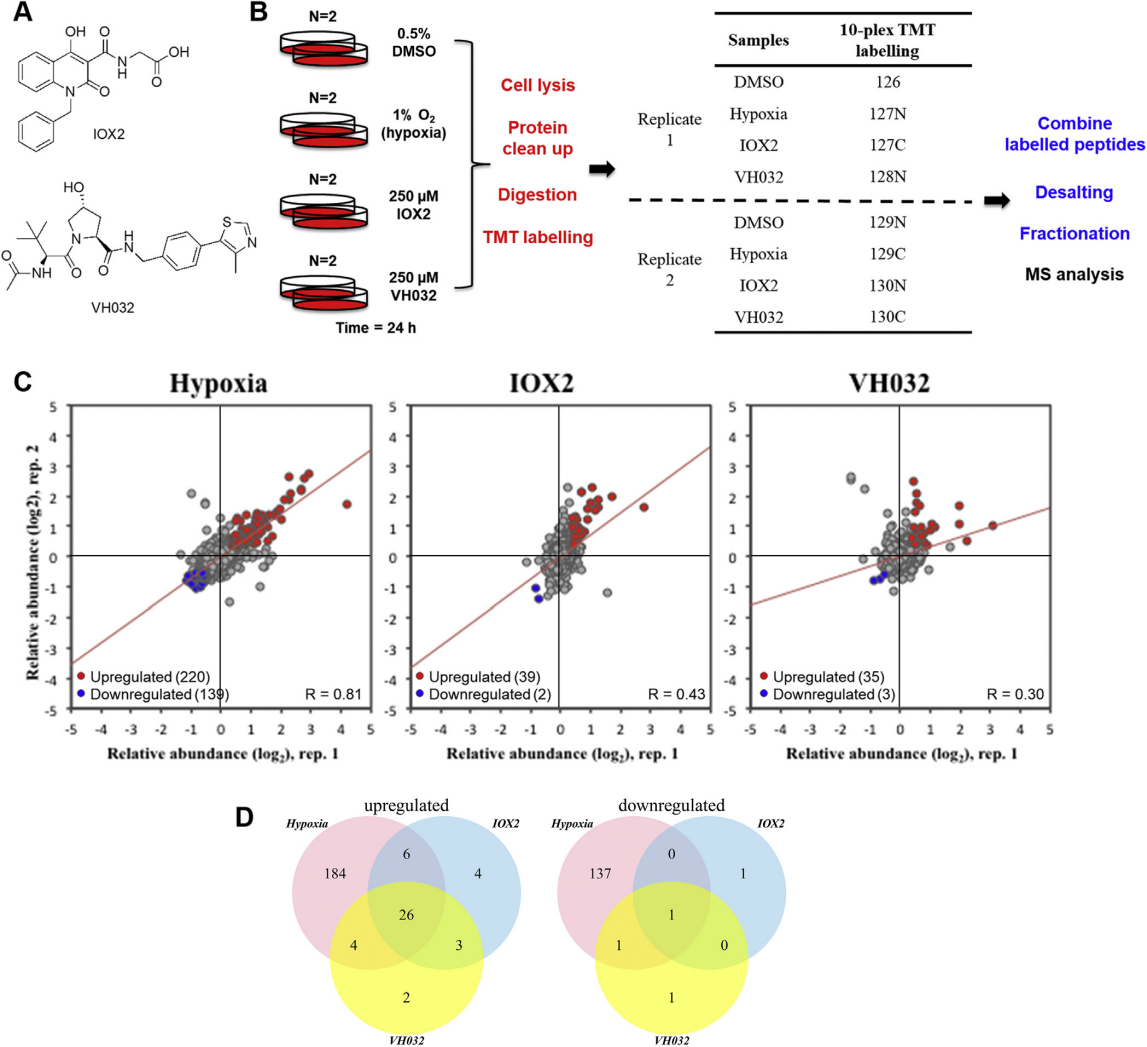
**Unbiased proteomics identify VHL protein level increase upon VHL inhibitor treatment.***A*, chemical structures of IOX2 and VH032. *B*, diagram depicting the workflow of tandem mass tag (TMT) labeling. HeLa cells were treated with 0.5% DMSO, 1% O_2_ (hypoxia), 250 μM IOX2, and 250 μM VH032 for 24 h—in biological duplicate. Proteins were obtained by cell lysis, cleaned up, digested by trypsinization, and labeled with 10-plex TMT labeling reagent. Labeled peptides were combined, desalted, fractionated, and analyzed by MS. *Red text* indicates steps carried out at the protein level, and *blue text* indicates steps performed on peptides. *C*, scatter plot representation of relative protein abundances obtained for different treatment conditions compared with respective replicate of vehicle (DMSO)-treated cells, for a total of 8043 proteins quantified. The two axes are relative abundance (log2FC) from two different replicates in this experiment. *Red dots* represent upregulated genes in both replicates (absolute fold-change difference to DMSO > 1.3), and *blue dots* represent downregulated genes in both replicates (absolute fold-change difference to DMSO < 0.7). *Red line* is the linear fit to the data. *D*, Venn diagrams depicting the number of upregulated genes and downregulated genes in the two replicates comparing the presence of hypoxia, IOX2, or VH032 to DMSO control. DMSO, dimethyl sulfoxide; VHL, Von Hippel–Lindau.

We have recently published our unbiased mRNA-Seq analysis of several HIF-stabilizing agents, including hypoxia, a broad-spectrum PHD inhibitor (IOX2), and a VHL inhibitor (VH032) (Fig. 1*A*) (16). In addition, we had characterized the cellular responses to a more potent and specific VHL inhibitor called VH298 (13, 14, 16). To gain a better understanding of the effect of inhibition of VHL by the small-molecule VHL inhibitor, global proteome analysis was performed in an unbiased manner. In a similar approach to our RNA-Seq analysis, HeLa cells were treated with 250 μM VH032, comparing with vehicle control (0.5% dimethyl sulfoxide [DMSO]), hypoxia (1% O_2_), and PHD inhibitor (250 μM IOX2). Enabled by the 10-plex TMT labeling–based strategy, a total of four conditions were performed in duplicate with the treatment time of 24 h, a time point long enough to observe the global proteome changes induced by HIF and any other changes elicited by the VHL inhibitor (Fig. 1*B*). By use of the MS–based method, more than 8043 proteins were identified with a false discovery rate <0.01 and quantified (Table S1).

We next determined the reproducibility between the two replicates by plotting the relative abundance for each protein to respective DMSO control between the two replicates (Fig. 1*C*). Hypoxia treatment showed the highest level of similarity in the proteins identified and quantified (Pearson correlation: 0.81); this was followed by IOX2 and VH032 treatments (Pearson correlation: 0.43 and 0.30, respectively). The mode of acquisition for the analysis is data dependent, which intrinsically generates variability within experiments (29). The lower correlation for the chemical inhibitors compared with hypoxia could be due to variability of inhibitor exposure across cells, but more work is needed to formally investigate this further. Analysis of the proteome changes identified revealed that the majority of upregulated proteins induced by VHL inhibitor were also identified in the hypoxia and IOX2 treatments, sharing 26 targets (Fig. 1*D* and Table 1). Of these, 22 genes were also identified in our RNA-Seq analysis, indicating changes to transcription vastly dependent on HIF (highlighted in Table 1). Of the additional four proteins shared by all these stimuli, notably HIF-1α is present, and other proteins are cholecystokinin, ryanodine receptor 3, and caspase recruitment domain family member 11.

**Table 1:** List of proteins upregulated in hypoxia, IOX2, and VH032 treatment

Uniprot ID	Gene name	Hypoxia replicate 1	Hypoxia replicate 2	IOX2 replicate 1	IOX2 replicate 2	VH032 replicate 1	VH032 replicate 2
*Q12983*	*BNIP3*	*18.11*	*3.33*	*11.59*	*2.6*	*8.33*	*2.03*
*P35318*	*ADML*	*6.47*	*4.73*	*2.05*	*4.81*	*1.47*	*3.44*
*O76061*	*STC2*	*3.24*	*1.59*	*1.58*	*4.36*	*1.84*	*1.31*
*P11169*	*GTR3*	*7.52*	*6.58*	*1.39*	*2.51*	*1.63*	*1.95*
*Q16790*	*CAH9*	*6.76*	*6.04*	*3.31*	*3.93*	*3.94*	*3.26*
P06307	CCKN	1.43	2.46	1.71	1.8	1.35	1.98
Q16665	HIF1A	4	2.31	2.37	3.68	2.19	1.98
Q15413	RYR3	2.32	1.87	2.2	2.86	1.45	4.3
*P98155*	*VLDLR*	*3.51*	*2.6*	*2.01*	*3.45*	*1.97*	*1.85*
*P05412*	*JUN*	*2.89*	*1.94*	*1.34*	*2.03*	*1.32*	*1.37*
*Q16877*	*F264*	*6.29*	*4.48*	*1.85*	*2.97*	*2.1*	*2.09*
*P11166*	*GTR1*	*3.59*	*2.79*	*1.35*	*1.72*	*1.47*	*1.42*
*Q9Y4K0*	*LOXL2*	*3.89*	*2.96*	*1.3*	*2.11*	*1.46*	*1.44*
Q9BXL7	CAR11	2.13	1.72	1.85	2.33	1.56	3.23
*Q9NX57*	*RAB20*	*2.5*	*1.93*	*1.44*	*2.34*	*1.58*	*1.49*
*P28300*	*LYOX*	*4.81*	*3.63*	*1.59*	*4.38*	*1.44*	*1.72*
*Q92597*	*NDRG1*	*5.06*	*4.25*	*1.36*	*1.58*	*1.62*	*1.41*
*B7ZBB8*	*PP13G*	*4.37*	*3.65*	*1.57*	*1.84*	*1.73*	*1.37*
*P13674*	*P4HA1*	*2.45*	*2.31*	*1.48*	*1.53*	*1.5*	*1.58*
*Q15118*	*PDK1*	*2.07*	*1.84*	*1.62*	*1.63*	*1.61*	*1.54*
*P09104*	*ENOG*	*3.03*	*2.62*	*1.39*	*2.23*	*1.7*	*1.74*
*Q9Y4C1*	*KDM3A*	*2.53*	*2.23*	*1.46*	*1.47*	*1.56*	*1.44*
*P27144*	*KAD4*	*1.42*	*1.38*	*1.31*	*1.38*	*1.33*	*1.37*
*O00469*	*PLOD2*	*2.76*	*2.53*	*1.41*	*1.5*	*1.55*	*1.58*
*Q9GZT9*	*EGLN1*	*1.87*	*1.61*	*1.37*	*1.44*	*1.39*	*1.34*
*Q9Y5U4*	*INSI2*	*2.19*	*2.18*	*1.31*	*2.39*	*1.32*	*1.54*

Proteins were selected at a false discovery rate <0.01 and relative abundance to control DMSO >1.3 for each replicate. Uniprot ID and gene name are listed with relative abundance to DMSO control. In italics: transcripts of these proteins were found upregulated in hypoxia, IOX2, and VH032 by RNA-Seq (16).

As expected, hypoxia induced the biggest changes in the proteome, including both induced and repressed protein expression (Fig. 1*D* and Table S2). This was also reflected in the mRNA-Seq analysis (16). In addition, more specific HIF inducers such as the VHL inhibitor and to some extent IOX2 broadly resulted in increased protein expression, with very few proteins found to be reduced in level (Fig. 1*D* and Table S2). This is in line with the knowledge that HIF is associated with gene induction and normally does not act as a transcriptional repressor (30).

### VHL inhibitors increase intracellular VHL protein levels

**Figure 2:**
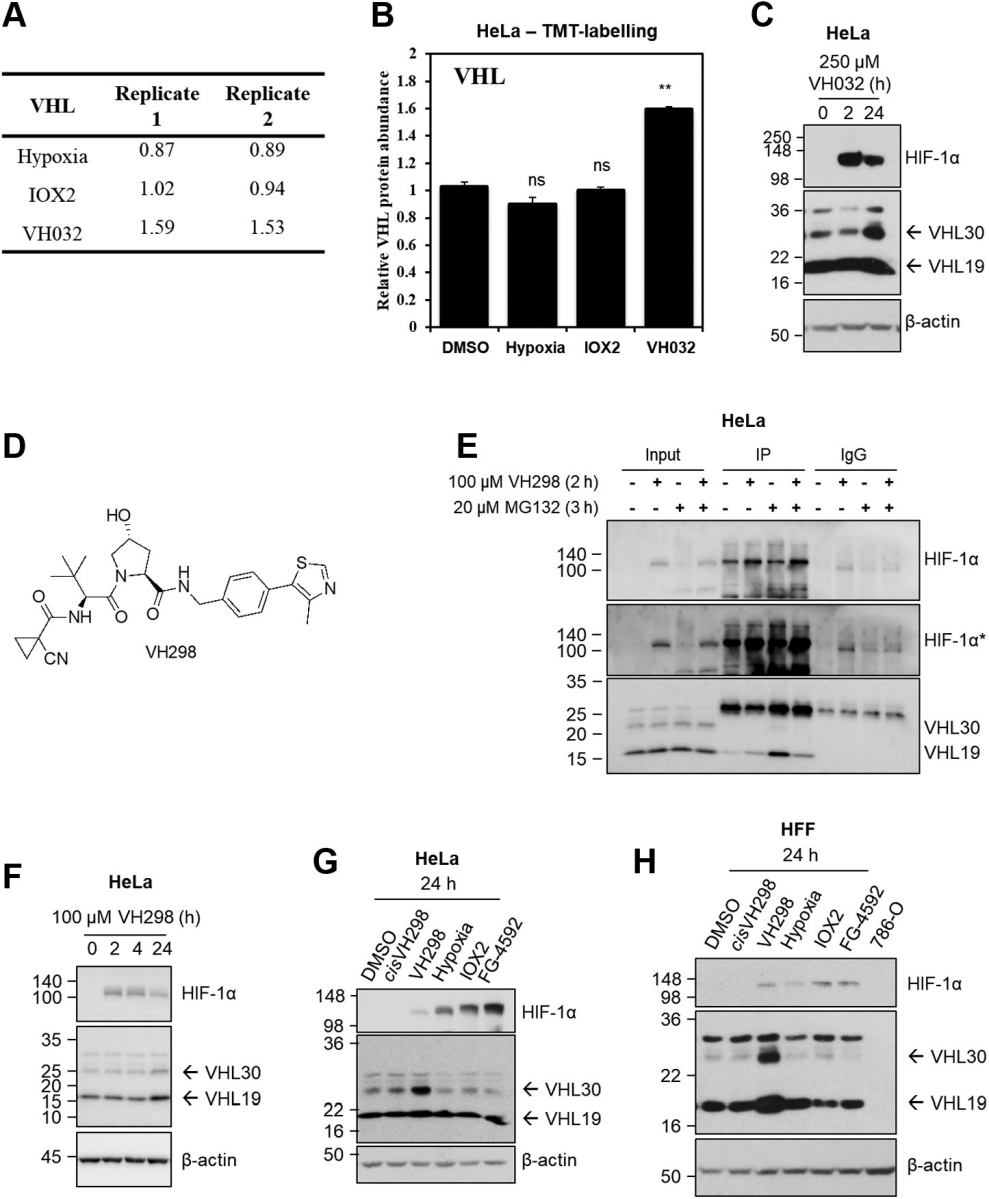
**VHL protein levels increase in the presence of VHL inhibitors.***A*, relative abundance and *B*, graph depicting relative VHL protein abundance with a false discovery rate <0.01 comparing with DMSO control for hypoxia, IOX2, and VH032 treatments after 24 h. *B*, data are presented as means + SD from proteomic analysis (TMT labeling) of two independent biological experiments. Two-tailed Student's *t* test was performed to calculate *p* values, and levels of significance are denoted as follows: ∗∗0.001 < *p* < 0.01 and, ns: *p* > 0.05. HeLa cells were treated with (*C*) 250 μM VH032 or (*F*) 100 μM VH298 for indicated time. *D*, chemical structure of VH298. *E*, coimmunoprecipitation on lysates from HeLa cells treated with vehicle DMSO (0.5% for 3 h), VH298 (100 μM for 2 h), MG132 (20 μM for 3 h), or VH298 and MG132 (100 μM VH298 for 2 h and 20 μM MG132 for 3 h) before lysis. About 300 μg of protein were used to immunoprecipitate with the 2 μg HIF-1α antibody (Santa Cruz; sc-53546). Mouse immunoglobulin G (IgG; 2 μg) was used as a control. Inputs represent 10% of the starting material used per immunoprecipitation (IP). *G*, HeLa or (*H*) HFF cells were treated with 0.5% DMSO, hypoxia (1% O_2_), and 100 μM of indicated compounds for 24 h. 786-O cell lysate was loaded in (*H*) as negative control for VHL bands. Protein levels were analyzed by immunoblotting using antibodies against HIF-1α, VHL, and β-actin, which acted as a loading control. HIF-1α∗ denotes longer exposure. The blots shown are representative of three independent experiments. DMSO, dimethyl sulfoxide; HFF, human foreskin fibroblast; HIF-1α, hypoxia-inducible factor-1α; VHL, Von Hippel–Lindau.

As we are particularly interested in the specificity of the VHL inhibitor, we turned our attention to the two proteins solely induced by VH032 compound: amylase 1 (AMY1) and VHL. AMY1 is a protein previously shown to be induced by hypoxia in plants (31), so strictly not associated with VHL inhibition only. We therefore turned our attention to VHL itself. VHL protein abundance increased upon VHL inhibitor treatment but not PHD inhibitor or hypoxia (Fig. 2, *A* and *B*). Relative to DMSO control, protein abundance of VHL increased to 1.59 for the first replicate and 1.53 for the second replicate (Fig. 2*A*). In contrast, VHL protein abundance was unchanged in IOX2 (1.02 for replicate 1 and 0.94 for replicate 2) and decreased slightly in hypoxia (0.87 for replicate 1 and 0.89 for replicate 2).

To validate the observation of the increase of VHL in the presence of VH032, we monitored VHL protein levels in HeLa cells treated with VH032 by Western blot. VHL protein levels did not increase after a short treatment of 2 h but showed marked increase in response to a longer treatment of 24 h with VH032 (Fig. 2*C*). In the course of the investigation, we identified and characterized a more potent VHL inhibitor, with enhanced cell permeability and cellular activity (VH298; Fig. 2*D*) that we qualified as a chemical probe (13, 14). Inside cells, VH298 completely disrupted the binding between HIF-1α and VHL, as shown in coimmunoprecipitation experiments (Fig. 2*E*). Therefore, we selected VH298 in our study moving forward. Like VH032, VH298 treatment resulted in increase in VHL protein levels (Fig. 2*F*), also in a time-dependent manner, confirming that this increase is due to the inhibition of VHL by the small molecules. In addition, the increase in VHL protein levels was not observed with the nonbinding epimer *cis*VH298 (Fig. 2, *G* and *H*), further demonstrating that accumulation of VHL results specifically from small-molecule binding to VHL. VH298 also increased VHL protein levels in another cell context, human foreskin fibroblasts (HFFs) (Fig. 2*H*). In both HeLa and HFF cells, the treatment of hypoxia or PHD inhibitor IOX2 did not increase VHL protein levels (Fig. 2, *G* and *H*), in agreement with the proteomic results (Fig. 2, *A* and *B*). We also confirmed that treatment with FG-4592, which is a more specific PHD inhibitor, showed similar results to those obtained with IOX2—namely no alteration in VHL protein levels. Altogether, VHL protein levels were confirmed to be increased by the small-molecule VHL inhibitors, not only in HeLa cells, in which the proteomic analysis was performed, but also in HFFs, and this increase was not observed following hypoxia or in the presence of PHD inhibitors.

### Ligand-bound VHL increases protein stability

**Figure 3:**
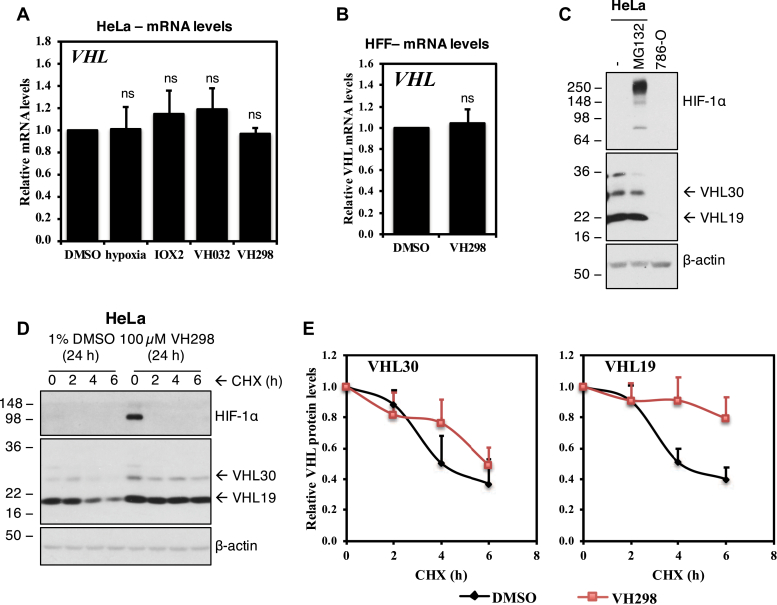
**VHL inhibitor stabilizes VHL proteins at a post-translation level.** VHL mRNA expressions in (*A*) HeLa cells treated with 0.5% DMSO, hypoxia (1% O_2_), and 250 μM IOX2, 250 μM VH032, or 100 μM VH298 for 16 h or in (*B*) HFF cells treated with 0.5% DMSO or 100 μM VH298 for 24 h. mRNA was collected, reverse transcribed, and analyzed by quantitative RT-PCR. The shown levels of the indicated mRNAs were normalized to those of β-actin. Graphs depict the mean + SEM of three independent biological replicates. Two-tailed Student's *t* test was performed to calculate *p* values, and levels of significance are denoted as follows: ns is *p* > 0.05. *C*, HeLa cells treated with 20 μM MG132 for 3 h. 786-O cell lysate was loaded as negative control for VHL and HIF-1α. *D*, half-life of VHL proteins of HeLa cells treated with DMSO negative control or VH298 VHL inhibitor was measured by treating cells with cycloheximide (CHX) for indicated times. *E*, protein levels of VHL30 and VH19 were quantified from multiple blots of different exposure time by ImageJ and plotted. Protein levels were analyzed by immunoblotting using antibodies against HIF-1α, VHL, and β-actin, which acted as a loading control. The blots shown are representative of three independent experiments. DMSO, dimethyl sulfoxide; HFF, human foreskin fibroblast; HIF-1α, hypoxia-inducible factor-1α; VHL, von Hippel–Lindau.

We next asked whether the increase in VHL protein levels in the presence of VHL inhibitor was due to the increase in mRNA levels. In quantitative RT-PCR assays monitoring *VHL* mRNA, mRNA levels of *VHL* were not altered in the presence of VH032 or VH298 in HeLa (Fig. 3*A*) or VH298 in HFF cells (Fig. 3*B*). Similar to the unaltered VHL protein levels, hypoxia and IOX2 did not induce changes in *VHL* mRNA levels (Fig. 3*B*). We next examined whether VHL might be autoregulating itself to be degraded by the proteasome, similar to how VHL regulates the proteasomal degradation of HIF-1α. However, results showed that VHL protein levels did not increase in the presence of proteasome inhibitor MG132 (Fig. 3*C*). We next asked whether the ligand-bound VHL might be more stable than the unbound form. We performed a cycloheximide chase experiment that inhibits *de novo* protein synthesis and monitored endogenous VHL protein levels to determine if the half-life of VHL increased in the presence of VHL inhibitor. Analysis revealed increased half-lives of both the long isoform of VHL and the short isoform (Fig. 3, *D* and *E*). *VHL* encodes two major VHL isoforms: a 213-amino acid isoform (pVHL_1–213_) and a 160-amino acid isoform (pVHL_54–213_) (32, 33). These two isoforms are also referred as pVHL30 and pVHL19, based on their apparent molecular masses upon protein electrophoresis. pVHL19 arises from an internal alternative translation initiation from Met54 within the open reading frame of VHL and thereby missing the N-terminal pentameric acidic repeat domain (33, 34). The VHL inhibitor VH298 increased the half-life of pVHL30 from 4 to 6 h, whereas the half-life of VH298-bound pVHL19 increased to beyond the 6 h cycloheximide treatment. This result correlates to the increased protein levels for pVHL30 and pVHL19 seen in the presence of VHL inhibitors (Fig. 2, *C* and *F*–*H*). Taken together, these data indicate that the binding of VHL inhibitor leads to increased VHL protein levels, as a result of protein stabilization, and delayed intracellular degradation.

### The VH298-induced VHL enhances proteasomal degradation of HIF-1α in prolonged VH298 treatment

**Figure 4:**
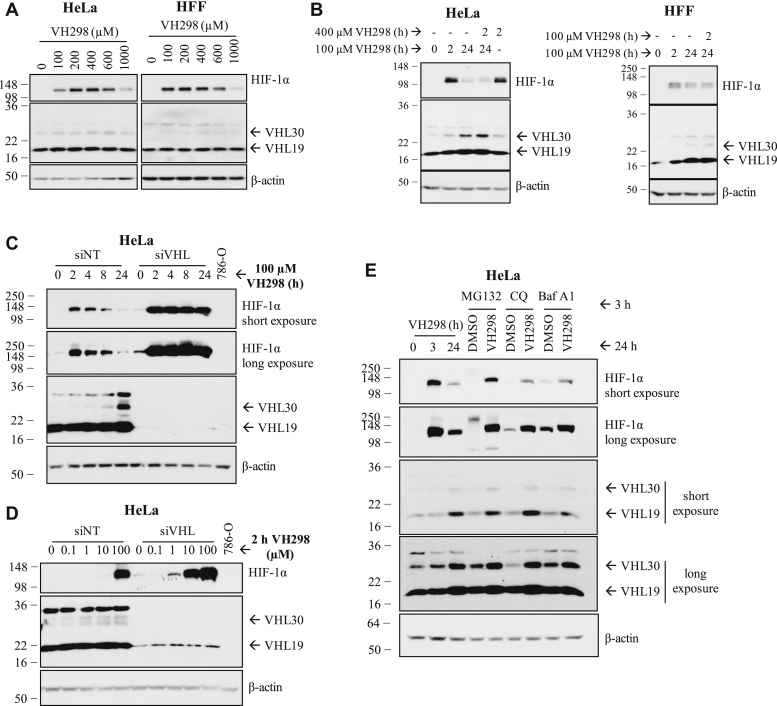
**The decrease of HIF-1α protein levels in prolonged VH298 treatment is mediated by proteasomal degradation in a VHL-dependent manner.***A*, dose-dependent immunoblots of HIF-1α in HeLa (*left panel*) and HFF (*right panel*) cells treated with VH298 for 2 h. *B*, HeLa and HFF cells were treated with 100 μM of VH298 for 2 and 24 h. After 24 h treatment of 100 μM VH298, 400 μM of VH298 was introduced to HeLa cells for 2 h and 100 μM of VH298 to HFF cells for 2 h. A 2 h treatment with 400 μM of VH298 was also included in HeLa cells. *C* and *D*, HeLa cells were transfected with nontargeting siRNA control (siNT) or VHL siRNA (siVHL). *C*, after 24 h, media were changed, and transfected cells were treated with 100 μM of VH298 for indicated times. *D*, after 46 h, media were changed, and transfected cells were treated with indicated concentrations of VH298 for 2 h. Cell lysate of 786-O was included as negative control for VHL. *E*, HeLa cells were treated with 3 and 24 h of 100 μM VH298. After 24 h treatments of 0.5% DMSO or 100 μM VH298, cells were treated with proteasome inhibitor MG132 (20 μM), autophagy inhibitors chloroquine (CQ; 50 μM) or bafilomycin A1 (Baf A1; 50 nM) for 3 h. Protein levels were analyzed by immunoblotting using antibodies against HIF-1α, VHL, and β-actin, which acted as a loading control. The blots shown are representative of three independent experiments. DMSO, dimethyl sulfoxide; HFF, human foreskin fibroblast; HIF-1α, hypoxia-inducible factor-1α.

We have previously observed that HIF-1α protein levels are acutely upregulated in the presence of VH298 because of inhibition of the VHL:HIF-α interaction, but this upregulation is not sustained in prolonged treatment and eventually decreased over time (Fig. 2*F*) (13). Since VHL protein levels increased in the presence of its inhibitor, we hypothesize that the decrease of HIF-1α levels observed in prolonged treatment of VHL inhibitor could be due to the increase of VHL protein levels. First, we investigated whether we could rescue the degraded HIF-1α by adding more VH298 to further inhibit the stabilized VHL. We determined the optimum concentration of VH298 to induce the maximum level of HIF-1α, and the doses were found to be 400 and 100 μM in HeLa and HFF cells, respectively (Fig. 4*A*). The optimum concentration of VH298 was then added to respective cells that had been treated with 100 μM VH298 for 24 h, at which point HIF-1α protein levels would have decreased. HIF-1α protein levels were monitored 2 h after the subsequent addition of VH298 (Fig. 4*B*). Although HIF-1α increased slightly, the addition of more VH298 did not rescue the degraded HIF-1α to the same level as the initially stabilized HIF-1α at 2 h in both HeLa and HFF (Fig. 4*B*) cells. These results could indicate that the decrease of HIF-1α levels in prolonged VH298 treatment was not because of insufficient VH298 to inhibit VHL. However, it is also possible that the extra-added inhibitor may be limited by cell permeability and so may still be insufficient to fully saturate intracellular VHL, particularly given the significantly increased VHL levels under those conditions. Therefore, we next investigated whether the VH298-induced HIF-1α is degraded in a VHL-dependent manner by performing siRNA-mediated knockdown of VHL in HeLa cells, followed by VH298 treatment. In control siRNA-treated cells, VH298 treatment gradually resulted in reduction of HIF-1α levels, as expected. In contrast, under VHL knockdown by siRNA, HIF-1α remained stabilized over the entire course of 24-h compound treatment (Fig. 4*C*). This suggests that the degradation of VH298-induced HIF-1α was indeed mediated by VHL. Interestingly, siRNA knockdown of VHL alone led to almost undetectable stabilization of HIF-1α (Fig. 4, *C* and *D*), as previously shown (28). This is consistent with siRNA knockdown not being able to completely remove all endogenous VHLs and confirms that even very low amount of the remaining VHL appears to be sufficient to efficiently polyubiquitinate HIF-1α for proteasomal degradation.

We postulate that the high activity and catalytic efficiency of VHL might explain the requirement of relatively high concentration of VHL inhibitor in order to observe detectable levels of HIF-1α. To examine this hypothesis, a dose-dependent VH298 treatment was performed with and without siRNA-mediated VHL knockdown (Fig. 4*D*). In the background of siRNA-mediated VHL knockdown, HIF-1α levels could be detected already at 1 μM concentration of VH298, in stark contrast to the negative control (siNT) background where HIF-1α levels were undetected at the same concentration of VH298 nor at 10-times higher concentration of VH298 (Fig. 4*D*). Remarkably, a 10 μM inhibitor treatment under VHL knockdown was found to achieve similar HIF-1α stabilization as 100 μM VH298 in the negative control siRNA background (Fig. 4*D*). The combination of VH298 and the knockdown of VHL showed additive effect on accumulating HIF-1α (Fig. 4*D*). Together, these data indicate that following treatment with VHL inhibitor VH298, HIF-1α is initially stabilized and then eventually degraded in a VHL-dependent manner, as VHL level increases.

HIF-1α is known to be degraded *via* the proteasomal (35) as well as lysosomal (36) pathways. To investigate the pathway involved in the degradation of VH298-induced HIF-1α, HeLa cells were first treated with VH298 for 24 h, followed by proteasome inhibitor (MG132) or lysosome inhibitors (chloroquine or bafilomycin A1) for the last 3 h. As expected, HIF-1α was stabilized upon each of the three inhibitors (Fig. 4*E*). After 24 h of VH298 treatment, MG132 rescued the degraded HIF-1α to similar protein levels as 3 h treatment of VH298 (Fig. 4*E*). In contrast, lysosomal inhibitors were not as efficient; both chloroquine and bafilomycin A1 increased HIF-1α to a much lower extent compared with MG132. Altogether, these data indicate that in prolonged VH298 treatment, the HIF-1α proteins that accumulated in the presence of VHL inhibitor VH298 are eventually degraded mainly *via* the proteasomal pathway, in a VHL-dependent manner.

## Discussion

In this study, we investigated the selectivity of a VHL inhibitor VH032 at the proteomic level. Using quantitative TMT labeling–based MS analysis, VH032 effects were compared with those elicited by hypoxia and the broad-spectrum inhibitor IOX2. This analysis complemented our previous mRNA-Seq analysis (16), as proteins that we identified changing were also captured at the mRNA level. This is important, as several global omics studies have reported a poor correlation of mRNA to protein (37). Furthermore, some transcripts are induced at different stages of the hypoxia response, and protein translation and stability might also be altered in hypoxia. Finally, we wanted to determine if the VHL inhibitor would have off-target effects at the protein level, not identified at the mRNA level. Our analysis confirmed that all HIF stabilizing agents used share an overlap of proteins that are upregulated. The vast majority of proteins identified as induced in our analysis overlapped with our mRNA-Seq as predicted. Furthermore, from the additional proteins identified, HIF-1α and known HIF targets were also present. This suggests that some HIF targets are induced at different times in the hypoxia response and that a more dynamic analysis of mRNA and protein would be required to fully cover all proteins induced by the stimuli used. Although VHL has been shown to control NF-κB activity (38, 39, 40), VH032 was found to target only the HIF response, as no NF-Κb–dependent signature was identified in our proteomic dataset or indeed in our RNA-Seq dataset (16). This further supports the specificity of the VHL inhibitor used in this study.

Given the widespread use of VHL inhibitors and VHL-based PROTACs as chemical tools to study biology, and as therapeutics, we were particularly interested in assessing target-specific effects and potentially identify any off-target effects. Our proteomics analysis revealed that VH032 is indeed exquisitely specific and selective for VHL. In fact, the only additional protein upregulated by VH032, not previously shown to be induced by hypoxia, was VHL itself, as AMY1 had previously been identified as a hypoxia-inducible protein (31). Stabilization of VHL was also confirmed by using the more potent inhibitor VH298. Further analysis revealed that this is dependent on VHL isoform stabilization and not alterations in mRNA levels. We have previously demonstrated direct binding of VH298 to VHL, which *in vitro* resulted in increased thermal stability both biophysically on the recombinant protein (41) as well as in a cellular environment (13). Interestingly, our results suggest that increased levels of VHL are responsible for reduced HIF-1α levels observed following prolonged exposure to VH298. Our data suggest that lowering the levels of VHL is able to circumvent the reduction of HIF-1α.

There are several implications for these observations. First, from a biological standpoint, it suggests that the HIF system explores increased VHL protein level as yet another negative feedback loop in place to prevent excess levels of the HIF transcription factor for prolonged periods. This adds to the known feedback loop of increased PHD2 and PHD3 protein levels (42). Second, it implies that changes to VHL levels can in itself regulate HIF-1α levels in cells. This is a concept that needs further exploring. Although proline hydroxylation is in most cases sufficient to maintain intracellular HIF-1α at low levels (3), increasing VHL levels can provide an additional mechanism to further reduce HIF-1α levels where necessary. Thus far, this mechanism remains understudied, and thus, future research is needed to investigate this further.

Our observations are also relevant to the pharmacology of VHL inhibitors such as VH298 or its derivatives as well as VHL-based PROTAC degraders. This is important given the wide use of these compounds as tools to study biology and the interest in their therapeutic development. VH298 has been shown to improve wound healing (18) and also tendon healing (17) in a rat model of injury. Other potential uses could be anemia and mitochondrial dysfunction, where HIF stabilization has been shown to be of benefit (43, 44). Our results demonstrating high target specificity of VH298 and feedback leading to reduced HIF-1α levels upon prolonged inhibitor treatment suggest that the use of a medicinal derivative of this chemical probe in these conditions could be highly recommended, as it would be predicted to have low side effects in its response. However, further analysis is now needed *in vivo* in these pathological conditions to firmly establish this possibility. With regard to PROTACs, to our knowledge, no study to date has reported of increased VHL levels upon PROTAC treatment. This may be in part because of the fact that VHL protein levels tend not to be monitored during PROTAC treatment, with the exception of homo-PROTACs and hetero-PROTACs aimed at inducing E3 ligase degradation (28, 45). It is also conceivable to imagine that VHL stabilization will not usually be observed because of the substoichiometric mode of action of PROTACs, working at sufficiently low concentration to induce target protein degradation, as opposed to the occupancy-based mode of action of inhibitors (19). Nonetheless, we advocate for the field to monitor intracellular protein levels of VHL when using VHL-based PROTACs. Interestingly, low intracellular expression of the E3 ligase VHL in platelets has been invoked to explain and exploit the observed reduced dose-limiting toxicity of VHL-based Bcl-xL PROTAC degraders (46).

We exemplify a case where a potent protein–protein interaction inhibitor can increase the intracellular levels of its target protein, while leading to a decrease in substrate recognition and hence enzyme activity. While this may not be unprecedented, we were able to find only one other such example in the literature: the enzyme thymidylate synthase, which is induced upon stabilization by its inhibitor 5-fluoro-2′-deoxyuridine 5′-monophosphate (47). When inhibiting metabolic or signaling pathways, enzyme target inhibition is often coupled to a feedback change in metabolite or substrate/product level, which in turn increases the transcription and/or the translation of the gene encoding for the enzyme target (48, 49). The net result is an increase in total target protein, leading to inhibitor/drug resistance. Other mechanisms of increase target level occur as a result of gene amplification (50) or release of the enzyme from binding to its own mRNA (51). Here, our results exclude transcriptional/translational upregulation and instead indicate that protein stabilization is the primary mechanism responsible for the increased intracellular levels of VHL. The finding that VHL inhibitors stabilize their target protein is consistent with current understanding of VHL structure and function. It is well documented that VHL acts as a tumor suppressor (6) and that the VHL protein has both moderate thermal stability (*T*_m_ ∼ 47 °C, see Refs. (13, 41)) and moderate cellular half-life (∼4–6 h, see Fig. 3*E* herein). We therefore hypothesize that VHL stabilization upon small-molecule binding leads to a reduction in intracellular degradation of VHL. We speculate that such protection away from degradation could be the result of oligomerization (to itself or partner proteins), by sequestration to other cellular compartments, or by stabilizing flexible and disordered regions on the VHL protein that normally confer its instability. Elucidating the detailed mechanism by which VHL inhibitors stabilize VHL warrants further mechanistic investigation in the future.

In conclusion, this work demonstrates the high specificity of VHL inhibitors VH032 and VH298 and that these inhibitors increase intracellular levels of their own target protein VHL, as a result of protein binding and stabilization. This has important implications for the pharmacology of VHL inhibitors and potentially VHL-based PROTAC degraders. We suggest that chronic long-term use of VHL inhibitors might benefit from our newly identified VHL stabilization, and therefore, pharmacological VHL blockade might not be as detrimental to cells or organisms as previously thought.

## Experimental procedures

### Cell culture and hypoxia induction

Human cervix carcinoma cells HeLa and HFF cells were obtained from American Type Culture Collection and propagated in Dulbecco's modified Eagle's medium (supplemented with 10% fetal bovine serum, l-glutamine, and 100 μg/ml of penicillin/streptomycin) at 37 °C. Cell lines were routinely tested for *mycoplasma* contamination using MycoAlert kit from Lonza.

### Hypoxia treatment

For hypoxia induction, cells were incubated at 1% O_2_ in an InVIVO 300 hypoxia workstation (Ruskin Technologies). In order to prevent reoxygenation, cells were lysed for protein extraction in the hypoxia workstation.

### Compound treatments

DMSO was used as vehicle control. The proteasome inhibitor MG132 was obtained from Merck/Millipore and used at the final concentration of 20 μM for 3 h. PHD inhibitors IOX2 and FG-4592 were purchased from Selleckchem. VHL inhibitors VH032, VH298, and nonbinding epimer *cis*VH298 were synthesized by a group member of our laboratory (13, 14). Compounds were added to cells for indicated length of time. Chloroquine (Merck) and bafilomycin A1 (Selleckchem) were added to cells for 3 h at 50 μM and 50 nM, respectively.

### siRNA transfections

siRNA oligonucleotides were purchased from Eurofins/MWG and used in a stock concentration of 20 μM. siRNAs were transfected using INTERFERin from Polyplus according to the manufacturer's instructions. The oligonucleotide sequences used for siRNA knockdown are the following ones:

siNT (nontargeting): 5′-AAC AGU CGC GUU UGC GAC UGG-3′

siVHL: 5′-GGA GCG CAU UGC ACA UCA ACG-3′

### Immunoblotting

Cells were lysed in radioimmunoprecipitation buffer (50 mM Tris, pH 8, 150 mM NaCl, 1% NP-40, 0.5% sodium deoxycholate, 0.1% SDS, 250 M Na_3_VO_4_, 10 mM NaF, and a protease inhibitor cocktail [Roche]) per 10 ml buffer. Proteins were resolved using SDS-PAGE, transferred onto polyvinylidene difluoride membranes in a semidry transfer system (BIORAD), and detected using primary antibodies, with β-actin as loading controls in mammalian cells.

Primary antibodies were used at following dilutions for mammalian cells: anti-HIF-1α (BD Biosciences; 610958; 1:1000 dilution), anti-VHL (Cell Signaling; #2738; 1:1000 dilution; product is discontinued), anti-VHL (Cell Signaling Technology; #68547; 1:1000 dilution), and anti-β-actin (Cell Signaling Technology; #3700s; 1:10,000 dilution). Following incubation with a horseradish peroxidase–conjugated secondary antibody (Cell Signaling), chemiluminescence (Pierce) was used for immunodetection.

### Coimmunoprecipitation

HeLa cells were lysed in radioimmunoprecipitation buffer as aforementioned. About 300 μg of cell lysates were used per immunoprecipitation condition. Protein lysates were incubated with 2 μg of HIF-1α antibody (Santa Cruz; sc-53546) or 2 μg of mouse IgG control antibody (Sigma) in a rotating platform at 4 °C overnight. About 20 μl of packed protein-G-sepharose beads (Pierce) were used to recover the immunocomplexes, by incubation in a rotating platform for 1.5 h at 4 °C. Beads were washed with 1× PBS buffer thrice. The complexes were eluted from beads with SDS-loading buffer and resolved as described previously by immunoblotting.

### Quantitative real-time PCR

For mammalian cells, RNA was extracted using the RNeasy Mini Kit (Qiagen) according to manufacturer's protocol. RNA was reverse transcribed using the iScript cDNA Synthesis Kit (Bio-Rad Laboratories). Real-time PCR was performed in triplicates using PerfeCTa SYBR Green FastMix (Quanta Biosciences) in C1000 Touch Thermal Cycler (Bio-Rad Laboratories). mRNA levels were calculated based on averaged cycle threshold values and normalized to β-actin mRNA levels.

Primer sequences used are listed herewith:

VHL: Forward, 5′-CCT TGG CTC TTC AGA GAT G-3′

VHL: Reverse, 5′-TGA CGA TGT CCA GTC TCC T-3′

β-actin: Forward, 5′-CTC TTC CAG CCT TCC TTC CTG-3′

β-actin: Reverse, 5′-GAA GCA TTT GCG GTG GAC GAT-3′

### Protein sample preparation and TMT labeling–based MS

HeLa cells were treated with 0.5% DMSO, hypoxia (1% O_2_), 250 μM IOX2, and 250 μM VH032 for 24 h. HeLa cells were lysed in 4% SDS in 100 mM Tris–HCl, pH 8.5 and 1× protease inhibitor (one cocktail tablet [Roche] per 10 ml of lysis buffer). Lysates were first sonicated for six cycles of 30 s on/30 s off under low-power setting with Bioruptor Twin (Diagenode) and then centrifuged at 17,000*g* for 10 min at 4 °C. Samples could be stored at −80 °C freezer. The experiments were performed in two biological replicates. The protein samples were further processed, including reduction, alkylation, digestion, desalting, TMT labeling, and fractionation according to the one previously described (24). TMT 10plex Isobaric Label Reagent Set (Thermo Fisher Scientific) was used despite two biological replicates of four conditions (a total of eight samples), as a duplicate of another condition was included in the MS that was not part of this project. The forth duplicate treatment was VHL inhibitor VH101, which however led to confounding the results because of its cytotoxicity, so is not included (14). Samples were labeled according to that indicated in Figure 1*A*.

### Nanoflow LC MS/MS analysis

Analysis of peptides was performed on a Q Exactive HF Hybrid Quadrupole-Orbitrap Mass Spectrometer (Thermo Fisher Scientific) coupled with a Dionex UltiMate 3000 RSLCnano Systems (Thermo Fisher Scientific). The peptides from each fraction were separated using a mix of buffer A (2% acetonitrile and 0.1% formic acid in Milli-Q water [v/v]) and buffer B (80% acetonitrile and 0.08% formic acid in Milli-Q water [v/v]). The peptides were eluted from the column at a constant flow rate of 300 nl/min with a linear gradient from 95% buffer A to 40% buffer B in 122 min and then to 98% buffer B by 132 min. The column was then washed with 95% buffer B for 15 min and re-equilibrated in 98% buffer A for 32 min. The Q Exactive HF Hybrid Quadrupole-Orbitrap Mass Spectrometer was used in data-dependent mode. A scan cycle comprised MS1 scan (*m*/*z* range from 335 to 1800, with a maximum ion injection time of 50 ms, a resolution of 120,000, and an automatic gain control value of 3 × 10^6^) followed by 15 sequential-dependant MS2 scans (with an isolation window set to 1.2 Da, resolution at 60,000, maximum ion injection time at 200 ms, and AGC 1 × 10^5^). To ensure mass accuracy, the mass spectrometer was calibrated on the first day that the runs are performed.

### Data processing and database searching

Raw MS data from 22 fractions were searched against Swiss-Prot database (March 8, 2015; *Homo sapiens*; 54,7964 sequences—after human taxonomy filter applied—20,203) using the MASCOT software (Matrix Science Ltd, version 2.2) through Proteome Discover software (Thermo Fisher Scientific, version 1.4). Mascot significance threshold was set to 0.05. Trypsin/P was specified as the cleavage enzyme allowing up to two missed cleavages. The database search included the following parameters: MS1 Tolerance: 10 ppm, MS2 Tolerance: 0.06 Da, fixed modification: Carbamidomethyl (C). Variable Modification: Oxidation (M), Dioxidation (M), Acetyl (N-term), Gln->pyro-Glu (N-term Q), hydroxyl (P), TMT10plex (N-term), and TMT10plex (K). The data were filtered by applying a 1% false discovery rate. Quantified proteins were filtered if the absolute fold-change difference between conditions to respective DMSO of the same biological replicate was ≥1.3 for upregulated proteins and ≤0.7 for downregulated proteins.

## Data availability

Data supporting the findings of this study are available within the article (and its supporting information files) and from the corresponding author upon reasonable request. The MS proteomics data have been deposited to the ProteomeXchange Consortium *via* the PRIDE partner repository with the dataset identifier PXD025743.

## Supporting information

This article contains supporting information.

## Conflict of interest

The Ciulli laboratory receives or has received sponsored research support from Almirall, Amphista Therapeutics, Boehringer Ingelheim, Eisai, Nurix Therapeutics, and Ono Pharmaceutical. A. C. is a scientific founder, advisor, and shareholder of Amphista Therapeutics, a company that is developing targeted protein degradation therapeutic platforms. The other authors declare that they have no conflicts of interest with the contents of this article.
